# PLCG2 and IFNAR1: The Potential Biomarkers Mediated by Immune Infiltration and Osteoclast Differentiation of Ankylosing Spondylitis in the Peripheral Blood

**DOI:** 10.1155/2024/3358184

**Published:** 2024-01-05

**Authors:** Bo Han, Qiaobo Xie, Weishi Liang, Peng Yin, Xianjun Qu, Yong Hai

**Affiliations:** ^1^Department of Orthopedics, Beijing Chao-Yang Hospital, Capital Medical University, GongTiNanLu 8#, Chao-Yang District, Beijing 100020, China; ^2^Joint Laboratory for Research and Treatment of Spinal Cord Injury in Spinal Deformity, Capital Medical University, Beijing, China; ^3^Clinical Center for Spinal Deformity, Capital Medical University, Beijing, China; ^4^Department of Orthopaedics, Capital Medical University, Beijing, China; ^5^Department of Pharmacology, School of Basic Medical Sciences, Capital Medical University, Beijing, China; ^6^Capital Institute of Pediatrics, Beijing 100020, China

## Abstract

**Objectives:**

Ankylosing spondylitis (AS) is a chronic inflammatory rheumatic disease characterized by chronic spinal inflammation, arthritis, gut inflammation, and enthesitis. We aimed to identify the key biomarkers related to immune infiltration and osteoclast differentiation in the pathological process of AS by bioinformatic methods.

**Methods:**

GSE25101 from the Gene Expression Omnibus was used to obtain AS-associated microarray datasets. We performed bioinformatics analysis using R software to validate different expression levels. The purpose of the GO and KEGG enrichment analyses of DEGs was to exclude key genes. Using weighted correlation network analysis (WGCNA), we examined all expression profile data and identified differentially expressed genes. The objective was to investigate the interaction between genetic and clinical features and to identify the essential relationships underlying coexpression modules. The CIBERSORT method was used to make a comparison of the immune infiltration in whole blood between the AS group and the control group. The WGCNA R program from Bioconductor was used to identify hub genes. RNA extraction reverse transcription and quantitative polymerase chain reaction were conducted in the peripheral blood collected from six AS patients and six health volunteers matched by age and sex.

**Results:**

125 DEGs were identified, consisting of 36 upregulated and 89 downregulated genes that are involved in the cell cycle and replication processes. In the WGCNA, modules of MCODE with different algorithms were used to find 33 key genes that were related to each other in a strong way. Immune infiltration analysis found that naive CD4+ T cells and monocytes may be involved in the process of AS. PLCG2 and IFNAR1 genes were obtained by screening genes meeting the conditions of immune cell infiltration and osteoclast differentiation in AS patients among IGF2R, GRN, SH2D1A, LILRB3, IFNAR1, PLCG2, and TNFRSF1B. The results demonstrated that the levels of PLCG2 mRNA expression in AS were considerably higher than those in healthy individuals (*P*=0.003). IFNAR1 mRNA expression levels were considerably lower in AS than in healthy individuals (*P* < 0.0001).

**Conclusions:**

Dysregulation of PLCG2 and IFNAR1 are key factors in disease occurrence and development of AS through regulating immune infiltration and osteoclast differentiation. Explaining the differences in immune infiltration and osteoclast differentiation between AS and normal samples will contribute to understanding the development of spondyloarthritis.

## 1. Introduction

Ankylosing spondylitis (AS), as a subtype of spondyloarthritis [1], is a chronic inflammatory rheumatic disease that is characterized by chronic spinal inflammation, arthritis, gut inflammation, and enthesitis [2]. Patients with fixed and kyphotic deformities caused by AS may face the problems such as impaired horizontal gaze, severe neck pain, and sagittal imbalance [3]. A combination of multistep surgery and digital planning is often required in complex AS deformities, which is a real challenge for spine surgeons [4]. AS is a considerable burden to patients and society because of deformity, pain, and disability [5].

Although the exact etiology and pathogenesis of AS are still unknown, mainly five hypotheses, including arthritogenic peptides, an unfolded protein response, human leucocyte antigen (HLA)-B27, homodimer formation, malfunctioning endoplasmic reticulum aminopeptidases, gut inflammation, and dysbiosis to explain the pathogenesis exist [6]. Genetic studies suggest that genetic factors account for about 90% of the pathogenesis of AS, and the genetic risk factors involved are major histocompatibility complex (MHC) and non-MHC gene loci [7]. Numerous studies have also shown that immune cell and osteoclast differentiation were crucial mechanisms and findings in the pathogenesis of AS [8–10].

Many studies focused on the key genes in the progression of AS by integrated bioinformatics analysis. However, there is no bioinformatics analysis related to the immune infiltration and osteoclast differentiation to analyze the function and regulation of differential genes. Hence, we conducted this investigation to identify the main biomarkers associated with immune infiltration and osteoclast development in the pathological process of AS utilizing bioinformatic techniques.

## 2. Materials and Methods

### 2.1. Data Download

**Figure 1:**
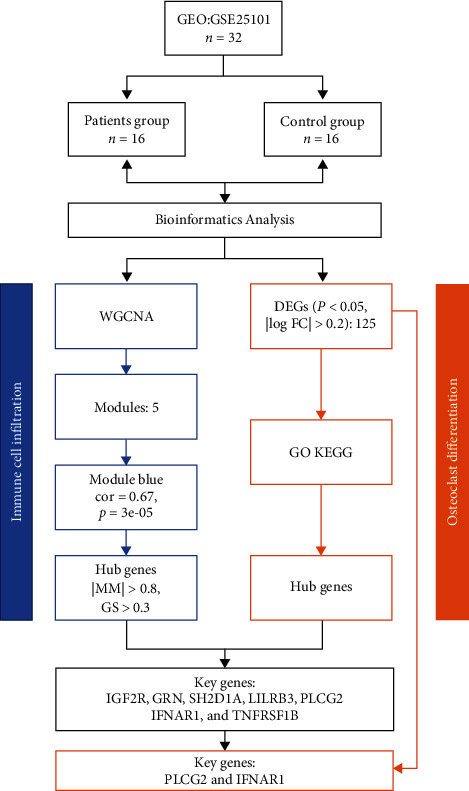
A flow-process chart shows the analysis steps in this study.

In our study, microarray data were retrieved from the Gene Expression Omnibus database (https://www.ncbi.nlm.nih.gov/geo/) [11] by using keywords “spondylitis, ankylosing” (All Fields) OR “Ankylosing Spondylitis” (all fields). The GPL6947 Illumina HumanHT-12 V3.0 expression bead chip serves as the foundation for this dataset. This chip comprises a total of 32 samples split between two groups. Under the comprehensive consideration, we finally selected GSE25101 as the research object. Patients' information and specimens' source of GSE25101 were outlined in Table 1. The dataset was obtained using R's “GEOquery” package (3.6.3) [12]. The workflow is shown in Figure 1.

### 2.2. Differential Expression Analysis

The “limma” package was applied to standardize and analyze patient and control data differences [13]. Differentially expressed genes (DEGs) were selected using the Benjamini–Hochberg adjusted *P* value <0.05.

### 2.3. Functional Annotation of DEGs

Gene ontology (GO) terms include biological process (BP), cellular component (CC), and molecular function (MF). The false discovery rate (FDR) <0.05 was significantly enriched. To reveal the function of the network, the “clusterProfiler” packages were used to perform GO and Kyoto Encyclopedia of Genes and Genomes (KEGG) analyses [14].

### 2.4. Construction of Weighted Gene Coexpression Network Analysis (WGCNA)

WGCNA is a systems biology approach that detects patterns of genetic linkage between diverse samples. Based on connectedness and relationship between genomes and phenotypes, it can reveal highly synergistic genomes, alternative biomarker genes, or therapeutic targets [15]. The “hclust” function was initially employed for hierarchical clustering analysis. Then, during module creation, we applied “pickSoftThreshold” to filter the soft thresholds and select the right power levels. To create the coexpression network, we employ the “WGCNA” program. Label each module with a distinct color, and then filter out the modules with the most interconnections. Using the “clusterProfiler” program and Metascape [16] (http://metascape.org), GO and KEGG analyses were performed on the genes in the modules. The screening criteria for crucial genes were gene significance (GS) > 0.70 and module membership (MM) > 0.80. We intersected the pivotal genes obtained from WGCNA with DEGs associated with osteoclast differentiation to obtain the essential genes. These genes make a significant contribution to the progression of AS.

### 2.5. Immune Infiltration Analysis

The immune cell infiltration matrix was produced by uploading gene expression profile data to CIBERSORT (https://cibersort.stanford.edu/) [17]. To visualize the differences in immune cell infiltration between control and patient groups, two-dimensional PCA clustering maps and violin plots were generated using the “ggplot2” software package. Heat maps of 22 infiltrating immune cells were generated using “pheatmap” (version 1.0.8) software [18]. Finally, differences in immune cell infiltration between the high and low-expression groups were analyzed and visualized according to the median expression levels of key genes.

### 2.6. RNA Extraction and Reverse Transcription and Quantitative Real-Time Polymerase Chain Reaction (qRT-PCR)

The peripheral blood was collected from six AS patients and six health volunteers matched by age and sex at the Beijing Chaoyang Hospital, Capital Medical University.

The inclusion criteria were as follows: (1) the patient was diagnosed with AS, (2) the age of patients ranging from 18 to 35-year old, (3) without osteoporosis or osteopenia, (4) early stage of the AS disease, (5) not associated with infectious diseases, and (6) the patient agrees and signs the informed consent form; The exclusion criteria were as follows: (1) medication was started, (2) with other rheumatic immune diseases, (3) with other chronic diseases, (4) with cancer in any system, (5) history of orthopedic surgery and diseases, and (6) no definite diagnosis of AS. All human specimens were acquired under the approval of the institutional review board of Beijing Chaoyang Hospital, Capital Medical University (2017-KE-67, Beijing, China). Under the guidance of the World Medical Association Declaration of Helsinki, we obtained informed consent from each patient.

The RNAprep Pure Hi-Blood Kit (Tiangen Biotech, China) was used to perform the reverse transcription of the extracted RNA. Using Nanodrop, the purity and amount of isolated RNA were evaluated. (Thermo Fisher Scientific, USA). cDNA was synthesized using reverse transcriptase (TIANGEN, Beijing, China). On an ABI 7500 Real-Time PCR System (Applied Biosystems), the SYBR Green Real-time PCR Master Mix (TOYOBO, Japan) was employed for quantitative PCR of hub genes. *β*-Actin was utilized as an internal control. All the primers (Sangon, China) used in this study are listed in Table 2. Normalization and calculation of relative mRNA expression were accomplished using the comparative Ct method (2^−*ΔΔ*Ct^). The data are shown as a fold change in expression relative to normal tissue. Comparisons were carried out through a one-way ANOVA, and *P* < 0.05 indicated that there were statistically significant differences.

### 2.7. Statistical Analysis

R software (3.6.3) was utilized to carry out the statistical analysis. Mean ± standard deviation (SD) was calculated for each and every set of numbers. Using the “limma” software tool, the difference in DEGs between control and the patients' groups were determined.

## 3. Results

### 3.1. Research Design Summary

Figure 1 shows the study's flowchart. Having screened for DEGs in AS using microarray data from the GEO database, we then screened for immune cells linked with AS using CIBERSORT. WGCNA and related techniques were utilized to identify genes focused on immune cells. The relationship between central gene expression and the clinical characteristics of AS was demonstrated using qRT-PCR.

### 3.2. DEGs between the Patients and Control Groups

**Figure 2:**
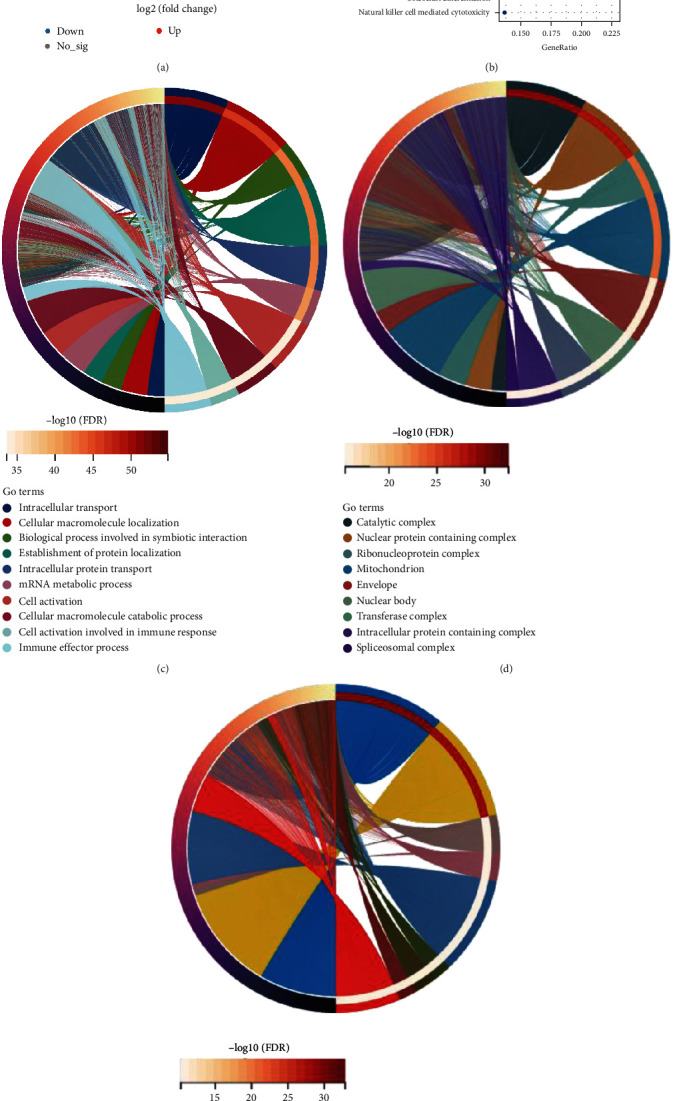
Functional annotation of DEGs. (a) DEGs were identified using a volcano plot, where red represents upregulated genes and blue represents downregulated genes. (b) KEGG pathway enrichment analysis for DEGs. (c–e) GO analysis of DEGs in (c) BP, (d) CC, and (e) MF.

The data on the expression profiles of the patients and the control group were compared and screened using a threshold of *P* less than 0.05 and a |logFC| value <0.2. We acquired a total of 125 DEGs, 36 of which were upregulated and 89 of which were downregulated among the genes (Figure 2(a)).

### 3.3. Enrichment Analysis of DEGs

The KEGG data revealed the enriched pathways for the related genes (Figure 2(b)). The findings of the GO analysis revealed that the DEGs were primarily abundant in BPs (Figure 2(c)). Intracellular transport, cellular macromolecule localization, the biological process involved in symbiotic interaction, and cell activation involved in immune response were among the substantially enriched BP keywords for the DEGs. Catalytic complex, nuclear protein-containing complex, and ribonucleoprotein complex were among the significantly enriched CC keywords for the DEGs (Figure 2(d)). Among the significantly enriched MF keywords for the DEGs, enzyme binding, RNA binding, and transcription factor binding were outlined as the first three (Figure 2(e)).

### 3.4. Immune Microenvironment Characteristics of AS

**Figure 3:**
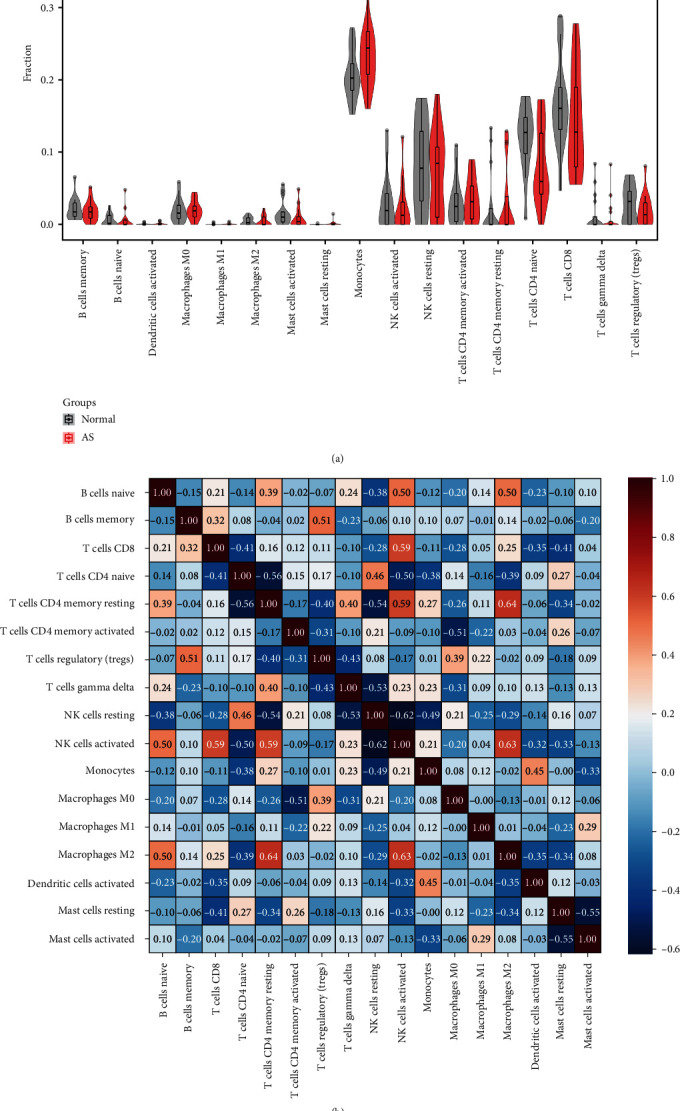
Immune infiltration landscape in whole blood. (a) Immune infiltration differences between patients and control group. (b) Correlation matrix of 22 immune cell type proportions. Some of the immune cells had a negative relation, denoted by the color blue, while others had a positive relation, represented by the color red. Compared to the lighter color, the association was much more robust (*P* < 0.05).

To gain a more comprehensive understanding of the immune environment present in AS, distinct immune cell types were analyzed using the CIBERSORT technique. After deleting cells with an immunological abundance value of “0,” the results showed that 17 different types of immune cells were examined, and it was found that the levels of naive CD4+ T cells and Monocytes were considerably higher in AS (*P* < 0.05; Figure 3(a)). Further research into the CIBERSORT scores revealed a strong positive association between B cells, Tregs, and M2 macrophages. This was illustrated by the correlation-based heatmap (corheatmap), which can be found in Figure 3(b). On the other hand, the infiltration of CD4+ T cells, NK cells, and M0 macrophages was found to relate to one another negatively. Together, as part of a joint process, the aberrant infiltration of immune cells seen in AS might have particular guiding relevance in the clinical management of the condition.

### 3.5. Identification of Immune Cell-Related Genes

**Figure 4:**
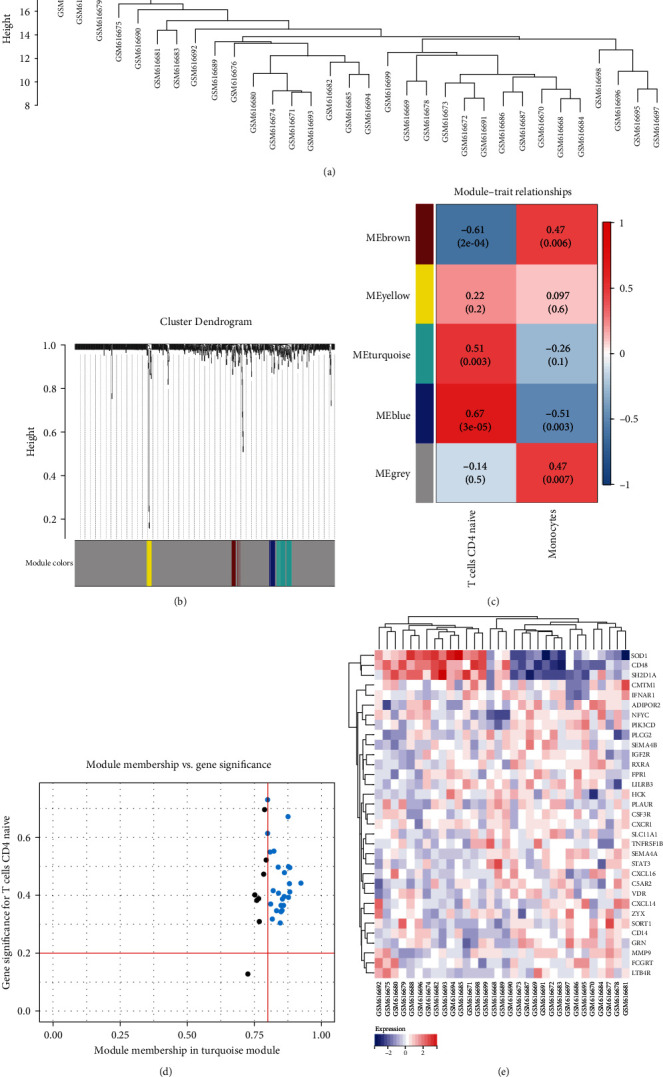
Identification of the immune infiltration-related genes in Ankylosing Spondylitis. (a) A clustering tree for the coexpression network module is created. (b) Feature of each combined module's relationship, with distinct colors denoting different parts. Each row represents a module. Each column shows how that module relates to the qualities, and each individual unit consists of both the *P*-value and the correlation coefficient. (c) Genes that belong to the blue module that was selected. (d) The blue portion of the collected genes. (e) A heat map of hub genes.

The WGCNA was applied to identify differentially expressed immune cell-related genes and explore the network's phenotype and hub genes (Figure 4(a)–4(c)). Twenty-five genes were selected by the correlation test. Cytoscape was used to create the interaction network that was comprised of these 25 genes as well as the genes that they were targeting. The WGCNA hub genes were intersected. Different colors were then used to differentiate these DEGs (Figures 4(b) and 4(c)). One of the four gene modules we assessed was tightly connected with immune cells (Figure 4(c)). The blue module had a strong positive correlation of 0.67 with naive CD4+ T cells, and *P* < 0.001 (Figure 4(d)). Based on the selection of the 30 most significantly elevated genes and the 33 most important genes, heat maps were generated (Figure 4(e)).

### 3.6. The Identification of Candidate Biomarkers

**Figure 5:**
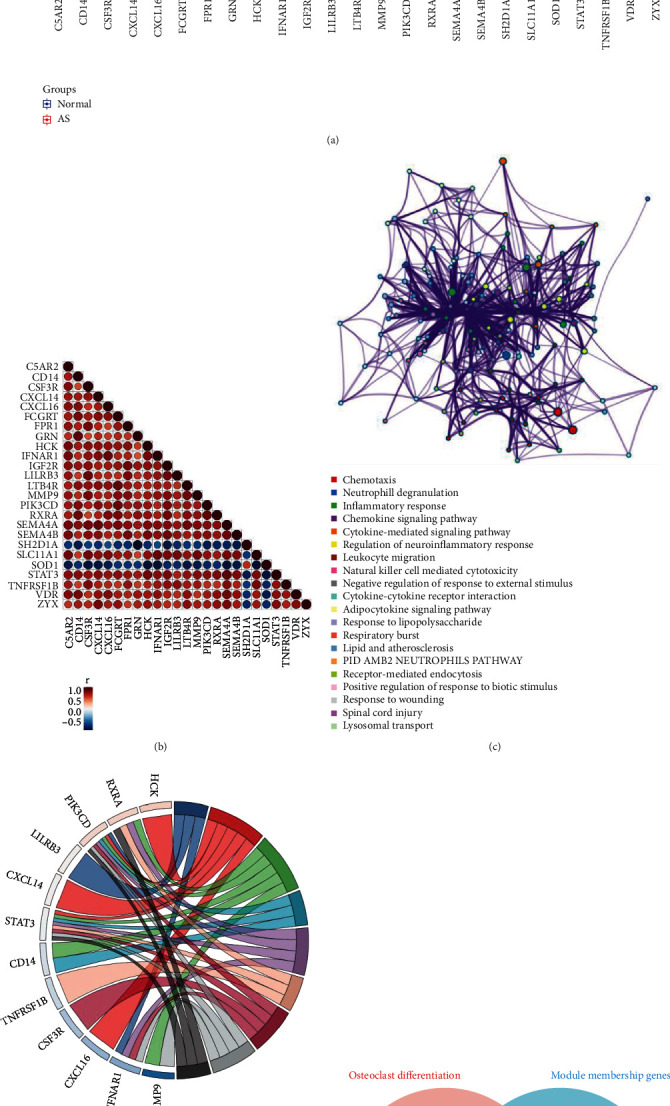
(a) The hub gene expression levels in different groups. (b) Matrix of expression correlations for each hub gene in AS. (c) The meta scale was used to enhance the functional enrichment of the correlated genes. Symbols “ ^*∗*^”, “ ^*∗∗*^”, and “ns,” respectively, stand for *P* < 0.05, *P* < 0.01, and nonsignificance. (d) Chord plots demonstrate key roles in hub genes. (e) Venn diagram showing the intersection of hub genes in the blue module in WGCNA and genes in the osteoclast differentiation signal pathway.

Twenty-five different gene modules were obtained due to the creation of the coexpression matrix (Figure 4(d)). Twelve genes satisfied the preselection criteria and were chosen based on relevant tests (Figures 5(a) and 5(b)). Finally, IGF2R, GRN, SH2D1A, LILRB3, IFNAR1, PLCG2, and TNFRSF1B were identified as key genes. After that, as depicted in Figures 5(c) and 5(d), we utilized metascape to collect more information on these genes and analyze their functions. Genes were enriched in essential biological processes, such as inflammatory response, chemokine signaling pathway, cytokine-mediated signaling pathway, regulation of neuroinflammatory response, leukocyte migration, and natural killer cell-mediated cytotoxicity.

According to the findings, most of the genes significantly enriched in pathways associated with immune response were the blue module.

We intersected the hub genes screened by WGCNA and the genes in osteoclast differentiation and obtained two genes: IFNAR1 and PLCG2.

### 3.7. qRT-PCR Validation of Data

**Figure 6:**
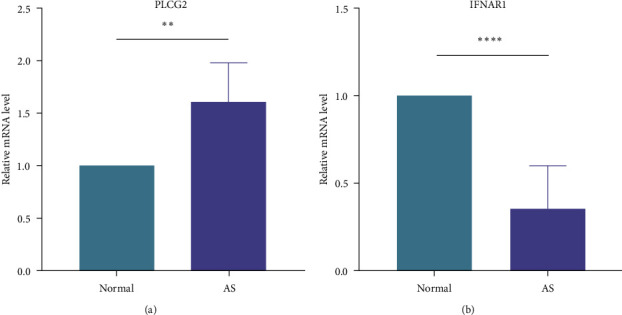
qRT-PCR validation in the peripheral blood. (a) The mRNA expression levels of PLCG2 in ankylosing spondylitis were significantly higher than that in the normal person (*P*=0.003). (b) The mRNA expression levels of IFNAR1 in ankylosing spondylitis were significantly lower than that in the normal person (*P* < 0.0001). (*P* < 0.05,  ^*∗∗*^*P* < 0.01, and  ^*∗∗∗∗*^*P* < 0.0001).

qRT-PCR experiments were performed to verify the bioinformatics results. The characteristics of patients and healthy volunteers are shown in Table 3. The results revealed that the mRNA expression levels of PLCG2 in AS were significantly higher than that in the normal person (*P*=0.003). The mRNA expression levels of IFNAR1 in AS were significantly lower than that in the normal person (*P* < 0.0001). All of the above results indicate that the outcomes of bioinformatics analysis are very competent and have considerable research value. (Figures 6(a) and 6(b)).

## 4. Discussion

AS is a common chronic inflammatory autoimmune disease in which axial inflammation, bone destruction, and new bone formation are the key events [19]. From 2005 to 2019 in China, the total prevalence of AS was 0.29%, among which there were 0.42% in males and 0.15% in females [20]. The mean AS prevalence was 0.24% in Europe, 0.17% in Asia, 0.32% in North America, 0.10% in Latin America, and 0.07% in Africa [21]. The most common symptoms of AS are chronic back pain and spinal stiffness, but peripheral and musculoskeletal manifestations are also frequently present [22].

In this study, we acquired a total of 125 DEGs, 36 of which were upregulated and 89 downregulated among the genes. IGF2R, GRN, SH2D1A, LILRB3, IFNAR1, PLCG2, and TNFRSF1B were identified as key genes enriched in the inflammatory response, chemokine signaling pathway, cytokine-mediated signaling pathway, regulation of neuroinflammatory response, leukocyte migration, and natural killer (NK) cell-mediated cytotoxicity. In terms of the inflammatory response, Guggino et al. [23] evaluated the activation and functional relevance of inflammasome pathways in AS patients and presented that inflammasomes drove type III cytokine production with an IL-1*β*-dependent mechanism in AS patients. The free heavy chain of HLA-B 27 may induce inflammation via T cells, NK cells, and bone marrow cells [24]. As is the case with AS, vascular endothelial cells respond to TNF by experiencing various pro-inflammatory alterations. The effectiveness of TNF-blocking medications in the treatment of AS demonstrates that TNF plays an essential part in inflammation [25]. In 17 different types of immune cells, we found naive CD4+ T cells and monocytes were considerably higher in AS. Zheng et al. [26] The AS contained a higher proportion of CD8+ T cells, naive CD4+ T cells, and neutrophils among CIBERSORT results. There was a strong positive association between B cells, Tregs, and M2 macrophages, while the infiltration of CD4+ T cells, NK cells, and M0 macrophages was found to relate to one another negatively. Zhang et al. [27] reported negative correlations in CD8+ T cells and neutrophils activated memory CD4+ T cells, which was similar to our results. Multiple immune cells control the activity of bone cells and the size of bones through the release of cytokines and signaling pathways. DCs and their subtypes play crucial roles in numerous autoimmune and chronic inflammatory diseases [28]. Increased plasmacytoid DCs have been found in the bone marrow and peripheral blood of AS patients, which has been linked to higher levels of inflammatory cytokines such as trafficking molecules, CCR6 and CCL20, TNF-, IL-6, and IL-23 [29]. The pathophysiology of AS can be better understood if the association between the immune and skeletal systems is further examined.

The phospholipase C gamma 2 (PLCG2) gene is responsible for encoding phospholipase C*γ*2 [30]. PLCG2 can regulate various cells' immune, inflammatory, and allergic responses through NFAT, NF-*κ*B, and MAPK signaling pathways [30, 31]. Yu et al. [32] found a point mutation in the mouse PLCG2 gene, which leads to hyperreactive external calcium entry in B cells and expansion of innate inflammatory cells, leading to severe spontaneous inflammation and autoimmunity. In humans, point mutations of PLCG2 can lead to autoimmune inflammation, resulting in arthralgia and inflammatory bowel disease, suggesting that point mutations of PLCG2 are an essential mechanism for inducing immune inflammation [33]. The destruction of bone and cartilage and local osteoporosis are important pathological manifestations of AS, among which osteoclasts play an essential role. Therefore, exploring the genes related to osteoclasts formation is of great significance. Studies have shown that PLCG2 mediator can induce osteoclasts while blocking PLCG2 enzyme activity can limit the development and function of early osteoclasts [34]. Normal bone remodeling requires a balance between the metabolic processes of bone-resorbing cells, osteoclasts, and bone-forming cells [35]. Jeong et al. [36] also found that betulinic acid could significantly inhibit the generation of osteoclasts by inhibiting the phosphorylation of PLCG2. Under the influence of promoting bone resorption factors, the multinucleated osteoclasts were formatted by the fusion and differentiation of monocyte progenitors, which could regulate osteoblast differentiation and bone formation [37].

IFN can stimulate the differentiation of immune cells and enhance immunological function, which may be an influential variable element in the pathogenesis of AS illness [38]. IFN can play an immunomodulatory role only when it binds to the interferon-*α*/*β* receptor (IFNAR). Studies have shown that IFN-*γ* polymorphisms are positively associated with the risk of AS [38]. Santiago-Raber et al. [39] found in NZB mice that a reduced number of IFN*α*/*β* receptors affected the incidence of immune lupus disease, suggesting the role of IFNAR in rheumatic diseases. However, the mechanism of IFNAR in the immune inflammation of AS remains unclear. It was reported that induction of IFN-B through the STING signaling pathway could restrai osteoclast differentiation and bone resorption, so it is speculated that decreased IFNAR can promote osteoclast differentiation [40].

In our study, PLCG2 and IFNAR genes were obtained by screening genes meeting the conditions of immune cell infiltration and osteoclast differentiation in AS patients. The above analysis indicated that inhibition of PLCG2 may inhibit the immune inflammatory response and osteoclast formation in patients with AS. In contrast, the increased expression of IFNAR may inhibit the immune inflammatory response and osteoclast formation.

Limitations of our study still remain. First, we used the GEO database instead of our patient data, and there was unknown bias such as duration of medication, the severity of AS, and race of patients. Second, data validation is not sufficient relatively and qRT-PCR should be used to show the link between hub gene expression and AS clinical characteristics. Based on our existing samples, we will subsequently integrate more samples and conduct in-depth research in peripheral blood, bone tissue, and single cells.

## 5. Conclusions

Dysregulation of PLCG2 and IFNAR1 are key factors in disease occurrence and development of AS through regulating immune infiltration and osteoclast differentiation. Investigating the differences between AS and normal samples in immune cell infiltration and osteoclast differentiation would contribute to a better comprehension of the root cause of spondyloarthritis and therapeutic methods.

## Figures and Tables

**Table 1:** Patients' information and specimens' source of GSE25101.

	Patient information				Source of specimen	
	Age (years, mean ± SD)	Sex		Family history	Tissue	Cell type
		Male	Female			
AS patients (*n* = 18)	45.9 ± 12.9	10	8	5	Whole blood	PBMC
AS control (*n* = 16)	NA	NA	NA	NA	Whole blood	PBMC

**Table 2:** PCR primers.

Gene	Forward primer sequence	Reverse primer sequence
IFNAR1	5′-TGTCCGCAGCCGCAGGTG-3′	5′-CCCGACAGACTCATCGCTCCTG-3′
PLCG2	5′-GGACATAGAGCTGGCTTCCC-3′	5′-GTTCAGTTCTTCTTGCCGCC-3′
Actin	5′-ACCGCGAGAAGATGACCCA-3′	5′-GGATAGCACAGCCTGGATAGCAA-3′

**Table 3:** The characteristics of patients and healthy volunteers.

Characteristic	Group AS	Group control	*P*
Age (yr)	30.2 ± 4.7	26.5 ± 4.8	0.21
Male sex-no. (%)	5 (83.33)	5 (83.33)	0.77
BMI (Kg/m^2^)	23.83 ± 4.17	23.17 ± 3.87	0.78
Race-Asian no. (%)	6 (100)	6 (100)	1
Positive for HLA-B27-no. (%)	6 (100)	/	
Time (Mons)	6.50 ± 3.27	/	/

## Data Availability

The data of this study are available from the corresponding author.
